# Response of earthworm enzyme activity and gut microbial functional diversity to carbendazim in the manured soil

**DOI:** 10.3389/fmicb.2024.1461880

**Published:** 2024-10-01

**Authors:** Tianyu Wang, Liping Zhang, Zhoulin Yao, Longfei Jin, Weiqing Zhang, Xianju Feng, Weibin Ma, Mei Lin

**Affiliations:** ^1^Key Laboratory of Fruit and Vegetable Function and Health Research of Taizhou, Zhejiang Citrus Research Institute, Zhejiang Academy of Agricultural Sciences, Taizhou, China; ^2^The Sainsbury Laboratory, University of East Anglia, Norwich Research Park, Norwich, United Kingdom

**Keywords:** manure, carbendazim, earthworm, enzyme activity, microbial functional diversity

## Abstract

The effect of pesticide pollution on environmental microorganisms in soil has become the focus of widespread concern in society today. The response of earthworm gut and surrounding soil microbial functional diversity and enzyme activity to carbendazim (CBD) was studied in a soil-earthworm ecosystem amended with manure. In the experiment, CBD was added to the manured soil (MS). Meanwhile, the pesticide treatment without manure and the control treatment without pesticides were also set up. The activities of catalase (CAT) and acetylcholinesterase (AChE) were measured to evaluate the toxicity of CBD. The Biolog method was used to assess the functional diversity of the microbial community. In the 2 mg/kg CBD treatment, earthworm AChE activity decreased significantly in the MS after 14 d, which occurred earlier than in the un-manured soil (NS). The changes of earthworm CAT activity in the pesticide treatments showed a trend of initially increasing and then maintaining at a high activity level. However, the CAT activities at 28 d in the manured soils were clearly lower than that at 7 d for both the CBD treatments, while they remained stable in the control treatments. The carbon source utilization, Simpson index, Shannon index, and McIntosh index of soil microorganisms in the MS treatments were significantly higher than those in the NS treatments. The overall activity of earthworm gut microorganisms in the MS treated with 2 mg/kg CBD was higher than that in the control. Also, CBD treatment (2 mg/kg) increased significantly the Simpson index and McIntosh index of earthworm gut microorganisms. The results indicated that the enzyme activities in the manured soils increased before 7 d for the pesticide treatments. Furthermore, exposure to CBD at a high concentration in the MS not only led to the earlier inhibition of earthworm enzyme activity but also significantly improved the overall activity of earthworm gut microorganisms and microbial functional diversity. This study revealed the ecotoxicological effects of earthworms in response to pesticide stress following the use of organic fertilizers under facility environmental conditions, which can provide a theoretical basis for the remediation of pesticide pollution in soil in the future.

## 1 Introduction

Earthworms are the most typical soil animals among temperate, tropical, and subtropical terrestrial ecosystems. They play an irreplaceable role in many key soil processes and are often referred to as “ecosystem engineers” (Datta et al., 2016; Liu et al., 2019). The swallowing and digging behaviors of earthworms play an important ecological role in the formation of soil structure, nutrient cycle, and pollution remediation (Wang et al., 2024; Liu et al., 2025). Many ecological functions of earthworms are related to their gut microorganisms. The digestive function of the earthworm gut can affect the structure and function of the soil microbial community (Blouin et al., 2013; Zhang et al., 2022). Earthworm gut microorganisms and soil microorganisms are important components of the soil ecosystem, which play an important role in element cycling, organic matter decomposition, pollutant degradation, improving soil fertility, and enhancing crop yield (Drake and Horn, 2007; Ma et al., 2016). However, pollutants such as pesticides, fertilizers, and heavy metals in the agricultural environment pose a threat to the ecosystem. As earthworms feed on organic matter in the soil, they come into contact with pollutants through their intestines, allowing these pollutants to enter into their bodies, where they accumulate in tissue and organs. This accumulation can produce certain negative effects on the survival, growth, and reproduction of earthworms (Liu et al., 2018; Qi et al., 2018; Wang et al., 2020).

In recent years, there has been increasing attention to the effect of adding exogenous organic fertilizer on soil fertility. There is a certain relationship between the use of organic fertilizer in soil and the microbial community structure in earthworms. Studies have shown that when various organic wastes in nature are fermented and contain a large number of microorganisms, which enter the digestive system of earthworms after being applied to the soil. Under the action of intestinal protease, lipase, fibrase, and amylase, organic matter is rapidly decomposed and transformed into nutrients that can be easily utilized by the earthworms themselves or other organisms (Van Groenigen et al., 2019; Vidal et al., 2023; Wu et al., 2024). After digestion by earthworms, the number of beneficial bacteria increases exponentially, while pathogen populations dependent on nutrient are controlled. Also, its excrement can play a role in promoting soil microbial structure and pollution remediation (Ferris and Tuomisto, 2015; Sułowicz et al., 2023; Chen et al., 2024).

At present, the effect of carbendazim (CBD) on earthworm enzyme activity and soil microbial functional diversity has been reported (Chuang et al., 2021; Guo et al., 2023; Kenko Nkontcheu et al., 2023; Zhou et al., 2023; Gautam et al., 2024). However, there are few studies focusing on earthworm enzyme activity and microbial functional diversity under the stress of CBD in manured soil (MS). Studies have shown that the stress of pesticides, heavy metals, and other pollutants can induce earthworm to produce reactive oxygen species (ROS) such as NO, H_2_O_2_, O_2_**·**^**−**^, and **·**OH^**−**^ (Yang et al., 2022; Xu et al., 2023; Lee et al., 2024; Yan et al., 2024). These ROS may activate the active oxygen scavenging system and lead to changes in some physiological and biochemical indices in earthworms (such as antioxidant enzyme system and non-antioxidant enzyme system). Such changes in these biochemical indicators can indicate pollutant toxicity and serve as an early warning system for soil pollution (Liu et al., 2017; Li et al., 2018; Zhang et al., 2021). Catalase (CAT) is a key enzyme in the biological antioxidant enzyme system, which defends organisms against antioxidant damage by catalyzing the decomposition of H_2_O_2_ into H_2_O and O_2,_ thus preventing cell peroxidation. Under the combined action of superoxide dismutase and CAT, O_2_**·**^**−**^ is eventually converted into H_2_O (Wu et al., 2012; Hu et al., 2016). Acetylcholinesterase (AChE) is a key enzyme in biological nerve conduction and can hydrolyze acetylcholine into choline and acetic acid to ensure the normal transmission of nerve signals (Calisi et al., 2013). CAT and AChE are sensitive to pollutants and are widely used to evaluate the pollutant toxicity.

The Biolog method is widely used in the study of environmental microbial ecology which is simple and does not require isolating and culturing microorganisms (Huang et al., 2024). This technique can obtain the metabolic fingerprint of the microbial community in a short time, characterize differences in physiological characteristics, and reflect the functional diversity of microbial community by measuring the variations in microbial utilization of different carbon sources (Gryta et al., 2014; Koner et al., 2022). This study will be more accurate, intuitive, and scientific in reflecting the effects of organic fertilizers on enzyme activities and microbial functional diversity in earthworms, in order to reveal the ecotoxicological effects of earthworms under pesticide stress from the perspective of microorganisms. Meanwhile, the results can provide reference and theoretical basis for the remediation of pesticide pollution in soil under facility environment.

## 2 Materials and methods

### 2.1 Chemicals and reagents

The protein quantitative determination kit (Coomassie brilliant blue method), acetylcholinesterase (AChE) test kit, and catalase (CAT) test kit (ammonium molybdate method) were provided by Nanjing Jiancheng Bioengineering Research Institute (Nanjing, China).

### 2.2 Experimental design and soil sampling

The sieved soils (3.5 kg dry weight) were weighed and then added 3% manure and stirred evenly. Then CBD standard solution was added to the MS to reach 1 mg/kg and 2 mg/kg corresponding to the recommended doses and the double dose, respectively (Daam et al., 2020). Meanwhile, sterile water was added to reach 60% of the soil’s maximum water holding capacity and the mixture was thoroughly stirred, passed the 2-mm sieve, and then transferred into plastic pots (upper diameter: 95 mm, height: 65 mm, bottom diameter: 70 mm). The mature earthworms (*Eisenia fetida*) were purchased from Shandong Agricultural University (Taian, China). Each plastic pot was filled with 150 g (dry weight) of soil, and 10 earthworms after clearing intestines were placed on the soil surface until they entered the soil actively. All treatments were performed in triplicate. Meanwhile, the pesticide treatments without manure and the control treatments without pesticide were set up including the un-manured control soil (NS-CK), the un-manured soil with 1 mg/kg CBD (NS-CBD1), the un-manured soil with 2 mg/kg CBD (NS-CBD2), the manured control soil (MS-CK), the manured soil with 1 mg/kg CBD (MS-CBD1), and the manured soil with 2 mg/kg CBD (MS-CBD2). All plastic pots were placed in the biochemical incubator at 20°C, 75% relative humidity, and 400 lux with a light–dark cycle of 12 h each. The soil water content was adjusted by weighing method every 2 d. After exposure to 1 d, 3 d, 7 d, 14 d, 21 d, and 28 d, 1–2 earthworms were taken out for the determination of enzyme activity. After exposure for 28 d, 5.0 g of soil sample and 0.3 g of earthworm gut tissue were collected for microbial functional analysis.

For earthworm gut dissection, several earthworms were taken out from each replicate and placed in a petri dish. The soil adhered to the earthworm surface was carefully removed. The earthworms were immersed in pure ethanol for 10 s to make them die quickly, and then washed with a 75% alcohol solution. In the clean bench, the earthworms were washed with sterile water 3 times and dried the earthworm’s surface water using the filter paper. Then the earthworm was fixed with pins on a foam board placed on filter paper and dissected with sterilized medical eye scissors. The body surface of the earthworm clitellum to the anus was cut off, and the gut tissue of the earthworm was carefully clamped out with sterilized tweezers, which was then weighed and placed into a 1.5 mL sterilized Eppendorf (EP) tube.

### 2.3 Determination of earthworm enzyme activity

Preparation of 5% earthworm tissue homogenate: 1–2 earthworms were taken from each treatment and placed in a petri dish padded with wet filter paper to clear the gut overnight. Nineteen times the volume of normal saline was added at the ratio of 1:19, homogenized in an ice water bath, centrifuged at 2500 rpm for 10 min, and the supernatant was taken for detection.

The enzyme activities were determined according to the manufacturer’s instructions for reagent kits (Jiancheng; China). The protein concentration of the sample was determined by the Coomassie brilliant blue method, and the protein content was determined based on the absorbance measured at 595 nm. The activity of CAT was calculated by measuring the absorbance of the light yellow complex formed by residual H_2_O_2_ and ammonium molybdate at 405 nm. The activity of AChE was detected by measuring the absorbance at 412 nm according to the color reaction of the TNB (symmetrical trinitrobenzene) yellow compound generated by choline and sulfhydryl chromogenic agents (Lin et al., 2016; Fang et al., 2022).

### 2.4 Microbial functional diversity in earthworm gut and soil

The overall functional diversity of microorganisms in the soil and gut after manured treatment was determined using Biolog® EcoPlates™ (Biolog Inc., Hayward, CA, United States) by referring to our previously used methods (Han et al., 2020). The metabolic potential of microbial communities was assessed using 31 different carbon sources categorized into six groups, including seven carbohydrates, 10 carboxylic acids, six amino acids, four complex carbon sources, two phosphate carbon sources, and two amines (Preston-Mafham et al., 2002; Ge et al., 2018; Nagata et al., 2023).

0.3 g of earthworm gut tissue was weighed into a 100 mL conical flask, and 30 mL of 0.85% NaCl solution was added and homogenized for 30 s. 5.0 g (dry weight) of soil sample was weighed into a 100 mL conical flask, and 45 mL of the sterilized 0.85% NaCl solution was added, shaken in a shaker under the conditions of 25°C, 150 rpm in the dark, and then placed in a clean bench to stand for 30 min. 1 mL of earthworm gut suspension or soil suspension was diluted to 10^−4^ diluents by the step-by-step dilution method. 150 μL of the diluent was inoculated into each well of the Biolog ECO plate with an 8-channel pipette. All plates were placed in an incubator at 25°C in the dark. After incubation for 4, 24, 48, 72, 96, 120, 144, and 168 h, all plates were read at 750 nm and 590 nm using a Biolog reader. All treatments were in triplicate.

### 2.5 Statistical analyses

Earthworm enzyme activity and microbial diversity index were analyzed by one-way analysis of variance (ANOVA) and carried out Duncan’s test with the IBM SPSS Statistics 26. The difference between OD590 and OD750 was used to represent the metabolic activity of microorganisms for subsequent data calculation and analysis. When the value was less than 0.06, it was treated as 0. The changes of microbial functional diversity index of earthworm gut and soil were analyzed by absorbance value of Biolog ECO plate after incubation for 72 h (Gryta et al., 2014; Fang et al., 2016; Urbaniak et al., 2024). The rate of substrate utilization by microorganisms was measured by calculating the average well color development (AWCD) using the following Equation 1:


(1) $$\mathrm{AWCD}=\sum \left(A-A_{CK}\right)/31$$

Where A represents the difference of absorbance value between two bands for each well in Biolog ECO plate, A_CK_ represents absorbance value of blank control well. Functional diversities assessed by the Shannon diversity index (H), Simpson index (1/D), and McIntosh index (U), were calculated using the Equations 2–4:


(2) $$H=-\sum \mathrm{pi}\left(\ln\;\mathrm{pi}\right)$$

(3) $$D=\sum \frac{\left(ni\left(ni-1\right)\right)}{\left(N\left(N-1\right)\right)}$$

(4) $$U=\sqrt{\left(\sum n_{i}^{2}\right)}$$

Where p_i_ represents the ratio of the relative absorbance value (A-A_CK_) of the well i to the sum of the relative absorbance value of the whole plate, n_i_ represents the relative absorbance value (A-A_CK_) of the well i, N represents the sum of the relative absorbance value of the whole plate.

## 3 Results

### 3.1 Effect of CBD on AChE activity

**Figure 1:**
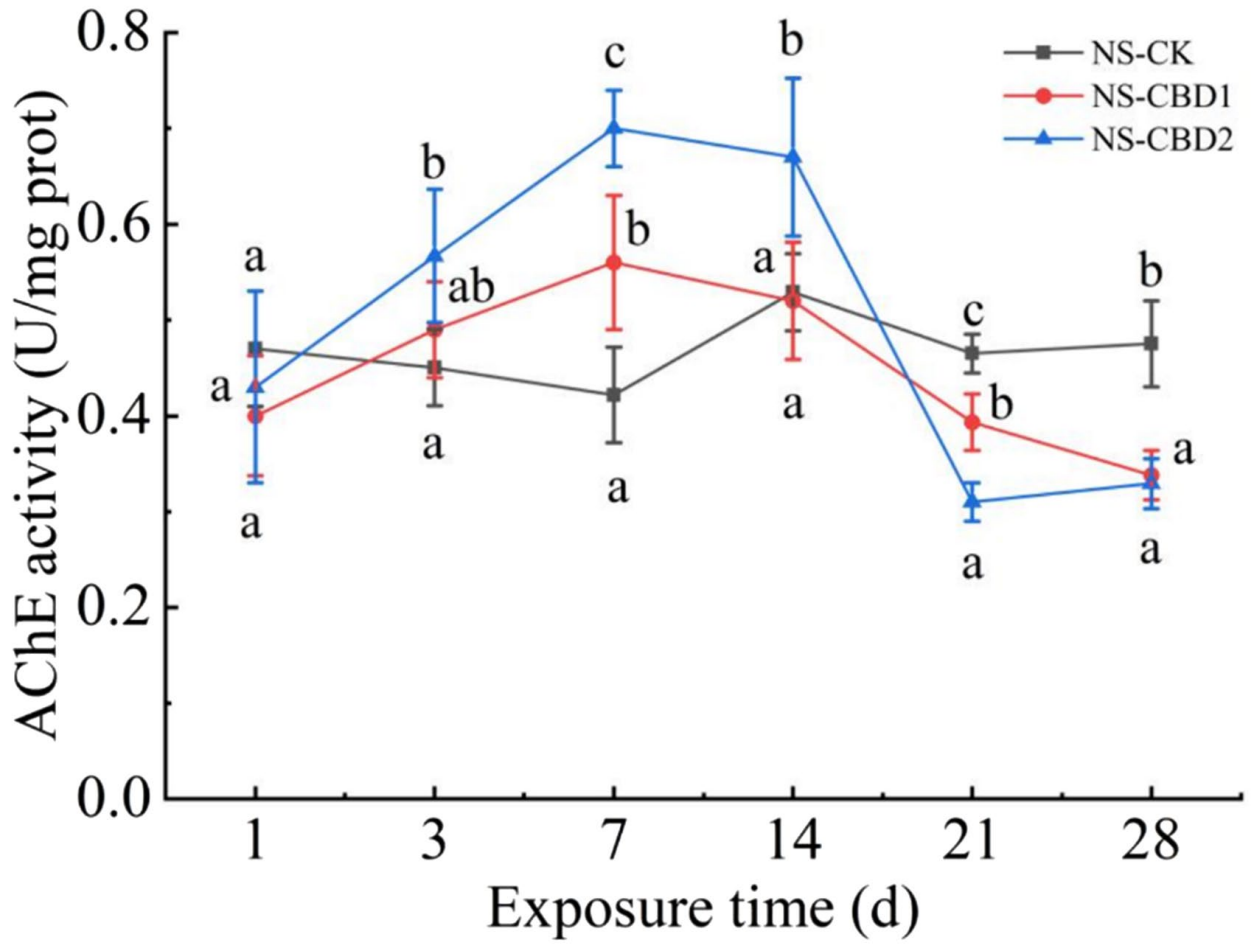
Effects of carbendazim on acetylcholinesterase activity of earthworm in the un-manured soil. NS-CK, NS-CBD1 and NS-CBD2 indicated 0, 1 and 2 mg/kg carbendazim treatment in the un-manured soil, respectively. The different letters above the curves indicate a significant difference (*p* < 0.05) based on variance analysis. The error bars represent the standard errors of the mean of triplicate samples.

**Figure 2:**
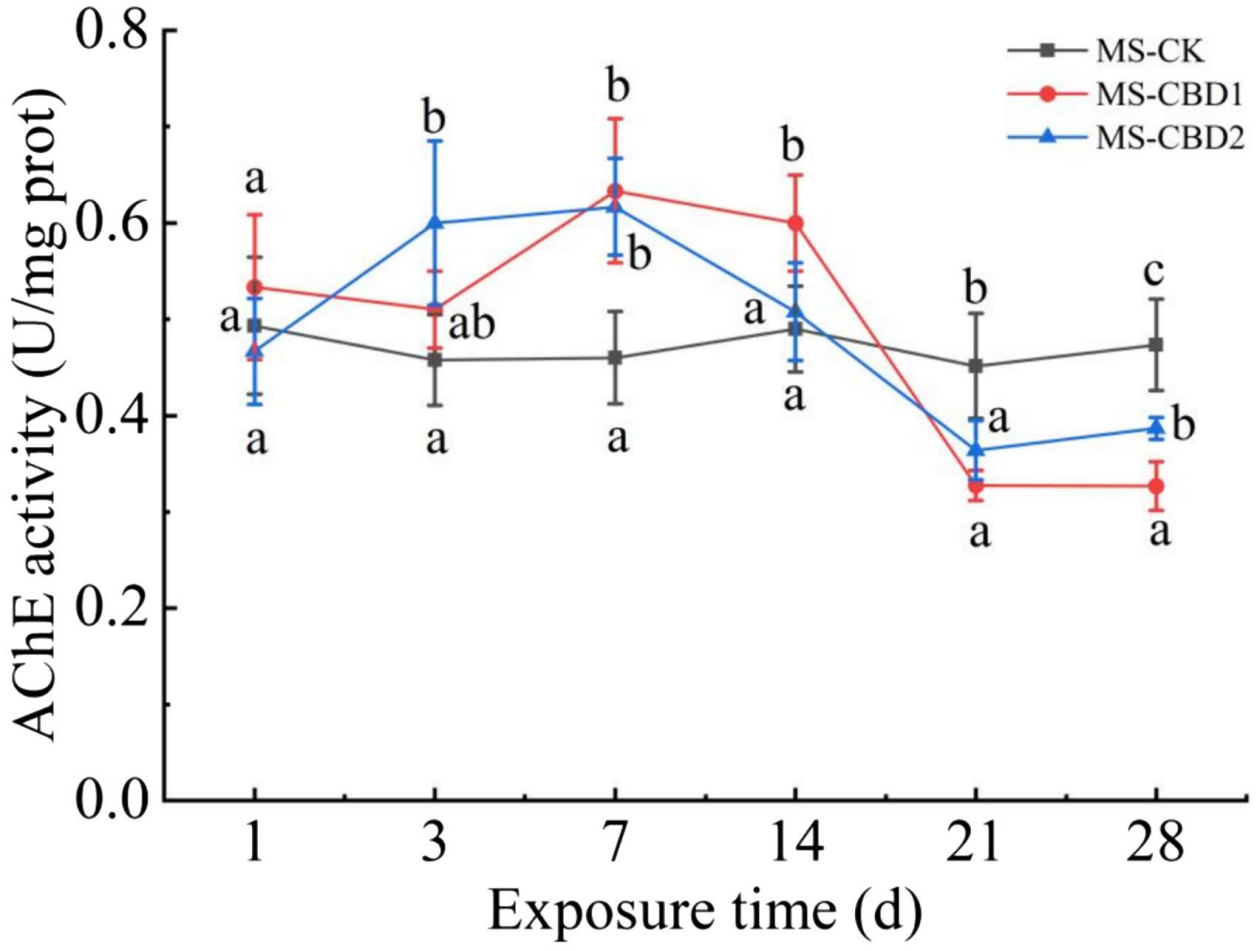
Effects of carbendazim on acetylcholinesterase activity of earthworm in the manured soil. MS-CK, MS-CBD1 and MS-CBD2 indicated 0, 1 and 2 mg/kg carbendazim treatment in the manured soil, respectively. The different letters above the curves indicate a significant difference (*p* < 0.05) based on variance analysis. The error bars represent the standard errors of the mean of triplicate samples.

The changes in acetylcholinesterase (AChE) activity of earthworms under CBD stress in un-manured soil are shown in Figure 1. In the control treatment (NS-CK), the AChE activity of the earthworms remained stable. CBD stimulated the AChE activity of earthworms at the initial stage (0 to 7 days) and significantly inhibited the AChE activity at the later stage (21 to 28 days) with the extension of exposure time. On the 7th day, the AChE activities of earthworms in NS-CBD1 and NS-CBD2 were at the highest, which were significantly higher than that of the NS-CK. On the 21th-28th days, the AChE activities of earthworms were significantly lower than those of the control. The changes in earthworm AChE activity in the manured soil are shown in Figure 2. CBD initially increased earthworm enzyme activity, which then subsequently decreased. MS-CBD2 was significantly higher than MS-CK on the 3rd and 7th days, while MS-CBD1 was significantly higher than MS-CK on the 7th and 14th days. In the later stage of exposure (21 to 28 d), however, earthworm AChE activities were significantly lower than those in MS-CK. The response trend of earthworm AChE activity under CBD stress in manured soil (MS) and un-manured soil (NS) is generally similar. The difference was that earthworm enzyme activity in MS-CBD2 treatment peaked on the 3rd day and decreased significantly on the 14th day, while in the NS-CBD2 treatment, it peaked on the 7th day and was significantly inhibited on the 21st day.

### 3.2 Effect of CBD on CAT activity

**Figure 3:**
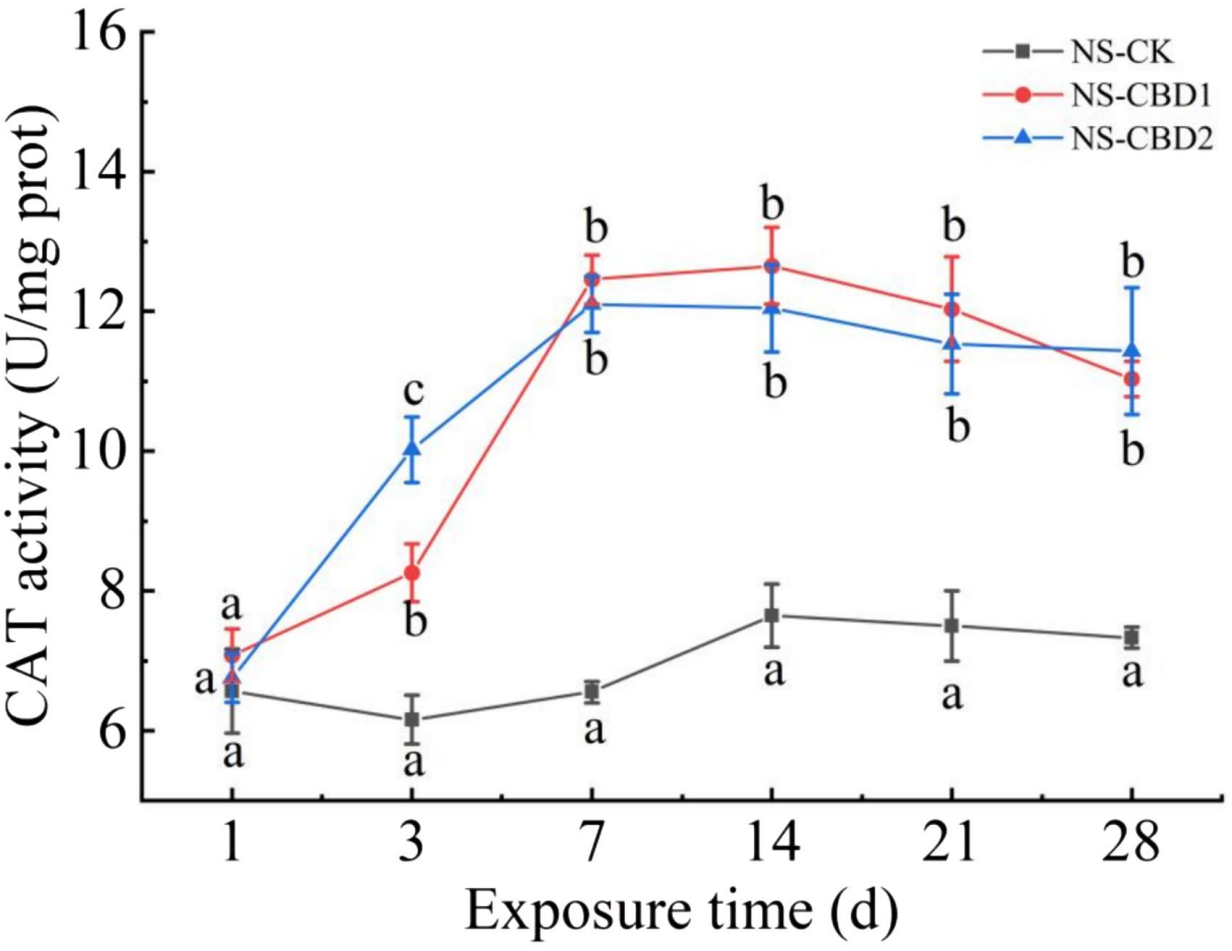
Effects of carbendazim stress on catalase activity of earthworm in the un-manured soil. The different letters above the curves indicate a significant difference (*p* < 0.05) based on variance analysis. The error bars represent the standard errors of the mean of triplicate samples.

**Figure 4:**
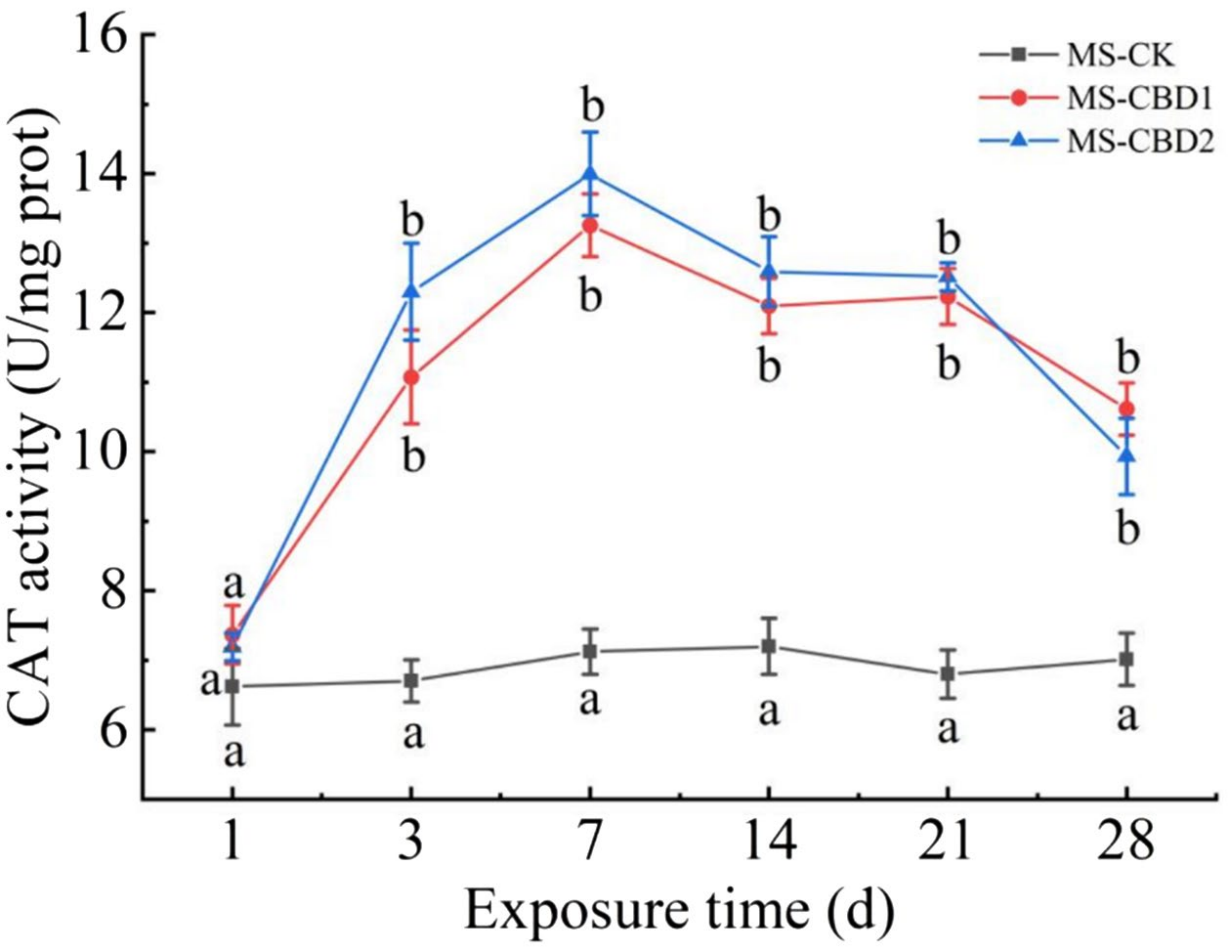
Effects of carbendazim stress on catalase activity of earthworm in the manured soil. The different letters above the curves indicate a significant difference (*p* < 0.05) based on variance analysis. The error bars represent the standard errors of the mean of triplicate samples.

The change of earthworm CAT activity under CBD stress in NS is shown in Figure 3. The CAT activities of earthworm in NS-CBD1 and NS-CBD2 were significantly higher than those in the NS-CK after 3 days. With the extension of exposure time, the CAT activity reached the highest level on the 7th day, and then decreased slowly within 7–28 days, but still maintained at a high level. The change of earthworm CAT activity in the MS is shown in Figure 4. The CAT activity of earthworms in the MS-CBD1 and MS-CBD2 increased significantly after 3 days, gradually reached to its peak after 7 days, and then slowly decreased between 14 to 28 days. Compared with MS-CK, CAT activity remained at a high level. In general, a similar trend was observed in both MS and NS: CBD treatment initially increased CAT activity and then maintained a high level. Furthermore, there were no significant difference in enzyme activity among different concentrations of treatment.

### 3.3 Effect of CBD on carbon source utilization diversity

**Figure 5:**
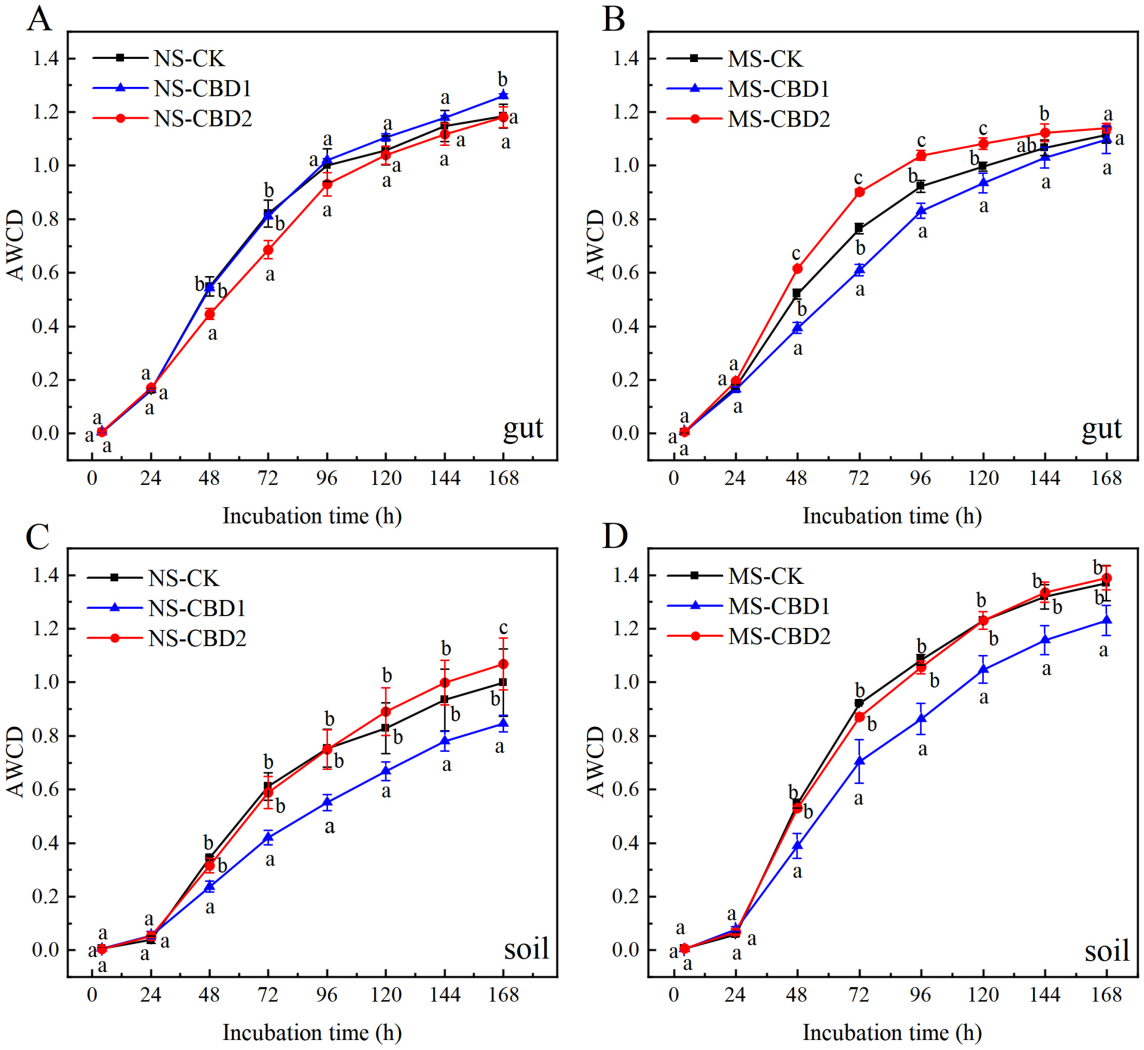
Variations in the average well color development (AWCD) of earthworm gut **(A,B)** and soil **(C,D)** microorganisms under different treatments. The different letters above the curves indicate a significant difference (*p* < 0.05) based on variance analysis. The error bars represent the standard errors of the mean of triplicate samples.

The changes of carbon source utilization diversity in earthworm gut and soil microorganisms under CBD stress are shown in Figure 5. In the MS and NS soils, the AWCD values of soil microorganisms in the 1 mg/kg carbendazim soil (CBD1) treatments were significantly lower than those in the control treatment, and the AWCD values of soil microorganisms in the 2 mg/kg carbendazim soil (CBD2) treatments were either slightly higher than or similar to those in the control soil. The AWCD values of soil microorganisms in all MS treatments were higher than those in the NS treatments. The AWCD values of earthworm gut microorganisms in the NS did not change significantly across all treatment groups, except for the NS-CBD2 where AWCD values were lower than the control at 48–72 h, but then returned to the control level. The AWCD values of earthworm gut microorganisms in the MS-CBD1 were lower than those of the control between 48 and 120 h, and then the activity gradually recovered to the control level. However, the AWCD values of earthworm gut microorganisms in the MS-CBD2 were not significantly different from those of the control at 0–24 h but were significantly higher than those of the control at 48–120 h, and then gradually recovered to the control level.

### 3.4 Changes in the functional diversity indices of microorganisms

**Figure 6:**
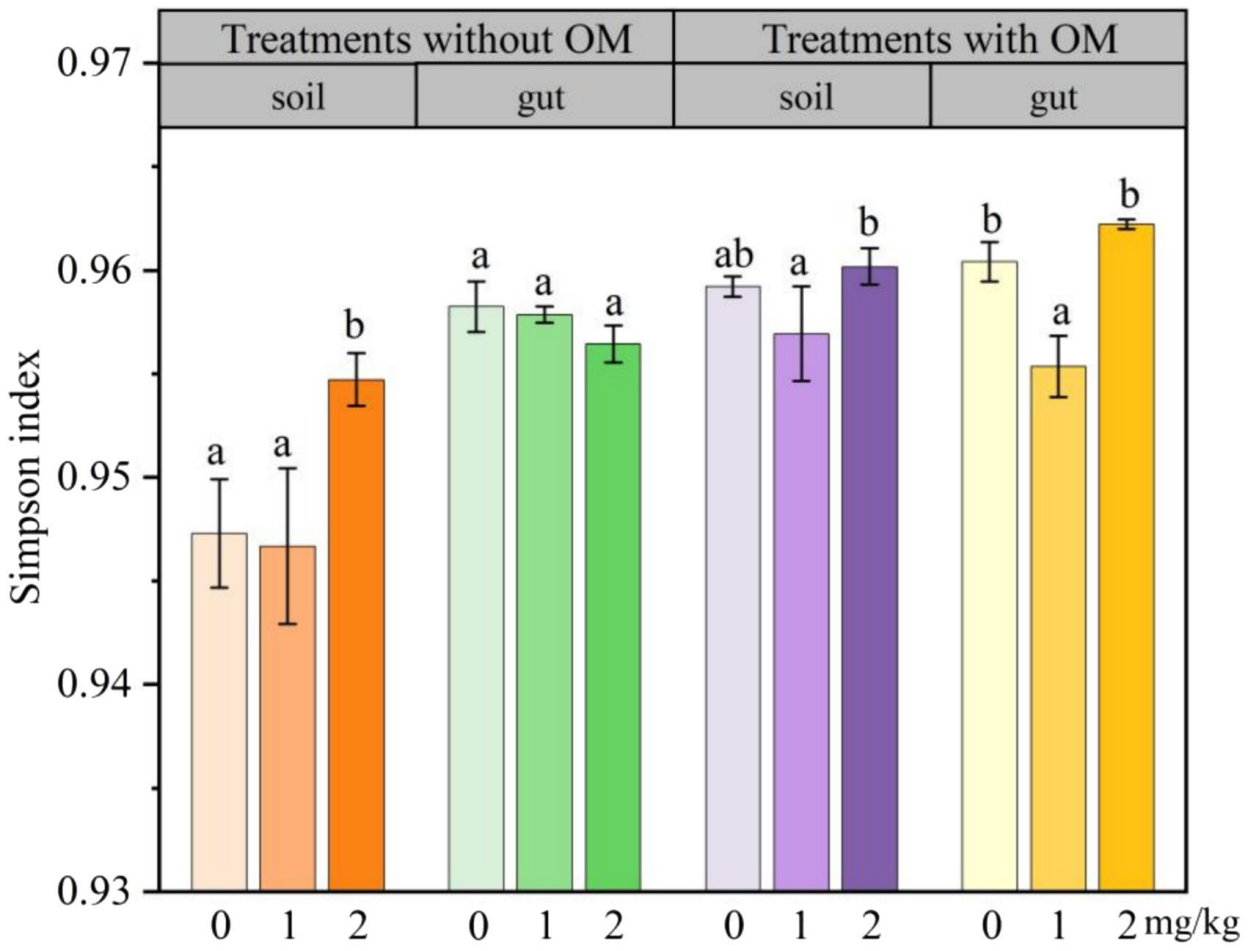
Changes in Simpson index of earthworm gut and soil microorganisms in different treatments. The different letters above the columns indicate a significant difference (*p* < 0.05) based on variance analysis. The error bars represent the standard errors of the mean of triplicate samples.

**Figure 7 fig7:**
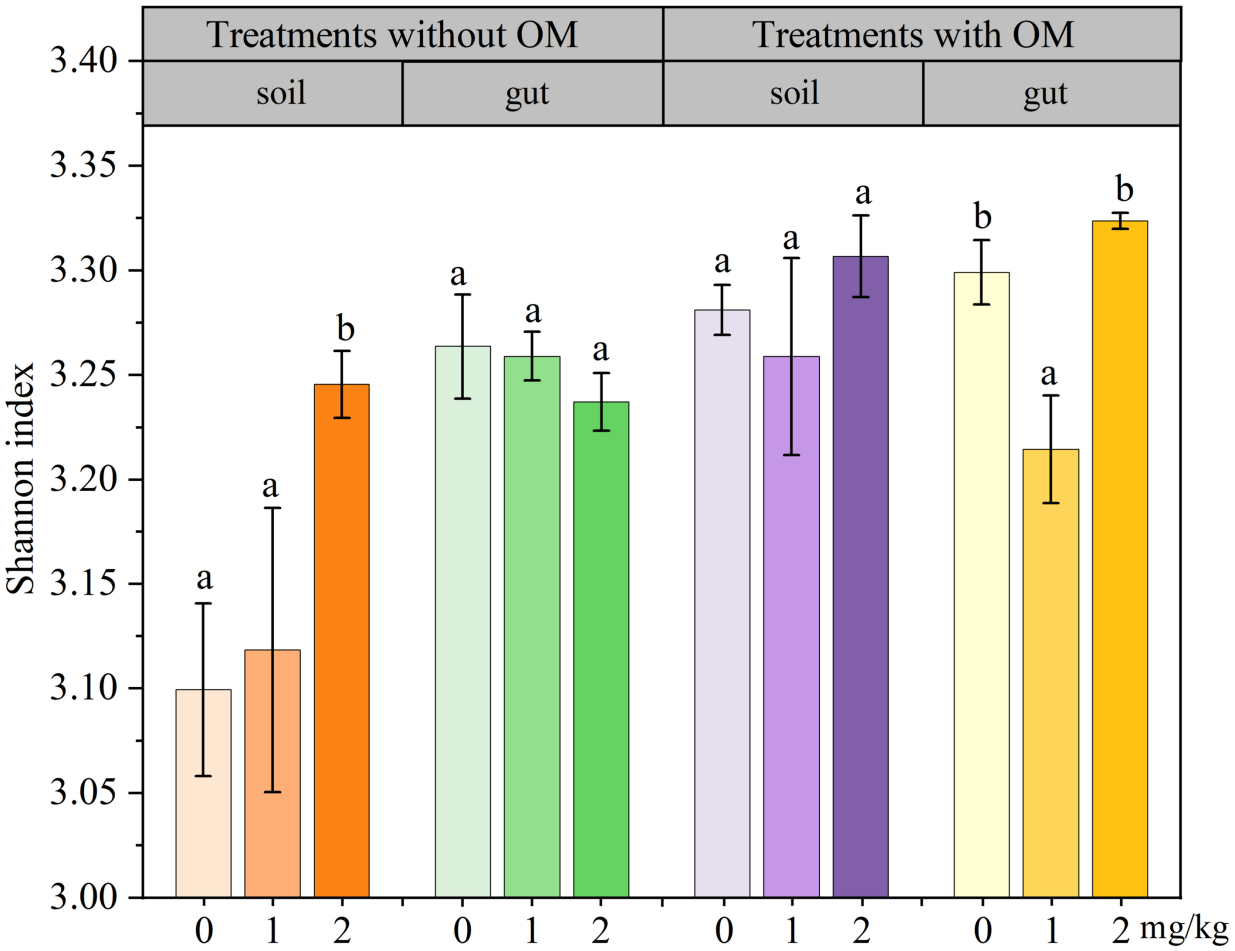
Changes in Shannon index of earthworm gut and soil microorganisms in different treatments. The different letters above the columns indicate a significant difference (*p* < 0.05) based on variance analysis. The error bars represent the standard errors of the mean of triplicate samples.

**Figure 8:**
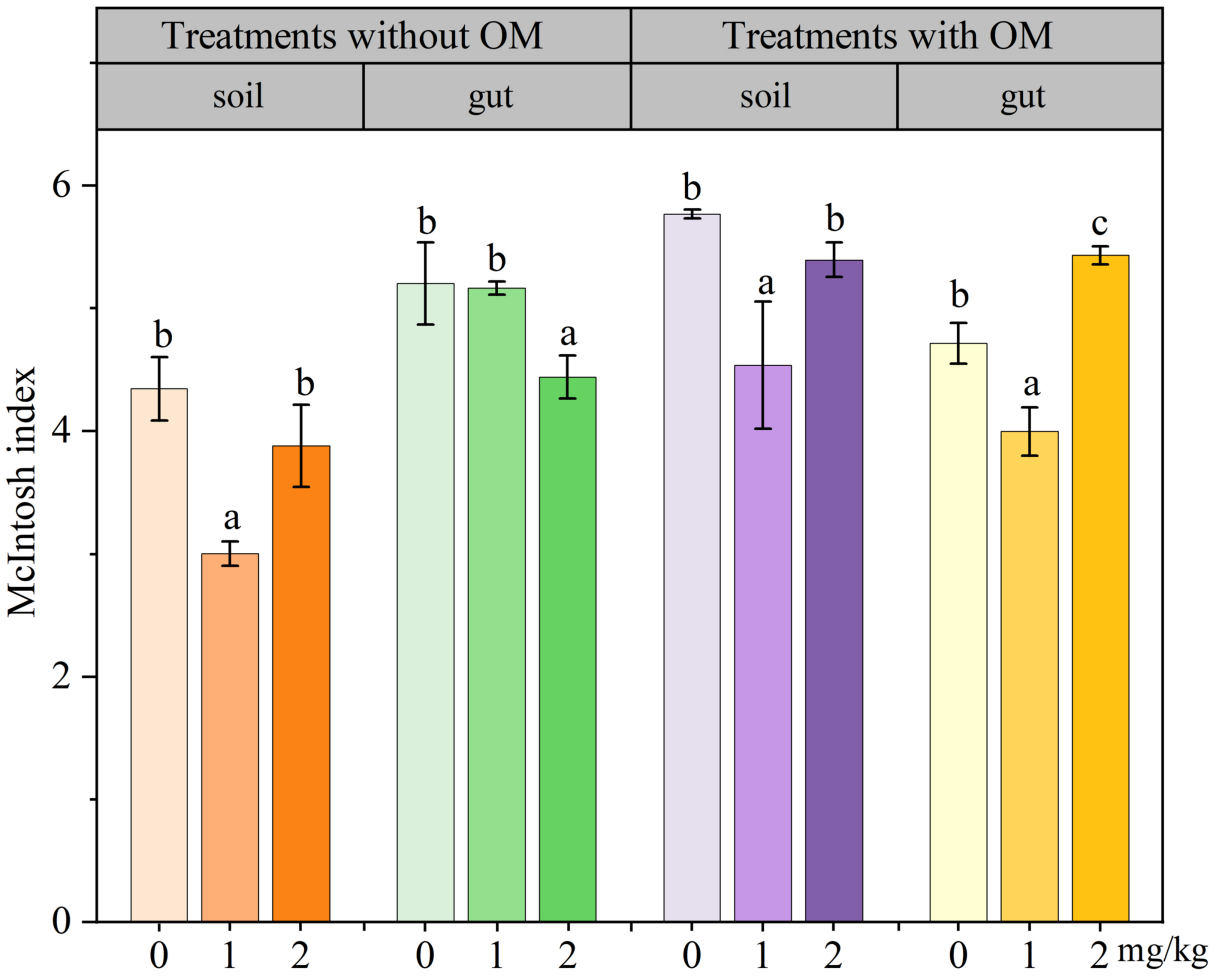
Changes in McIntosh index of earthworm gut and soil microorganisms in different treatments. The different letters above the columns indicate a significant difference (*p* < 0.05) based on variance analysis. The error bars represent the standard errors of the mean of triplicate samples.

The changes in soil microbial functional diversity indices under CBD stress are shown in Figures 6 [Fig fig7]–8. The 1/D and H of soil microorganisms in the NS-CBD2 were significantly higher than those in the NS-CK, while the U showed no significant difference compared with the NS-CK (*p* < 0.05). In the NS-CBD1, there was no significant difference between the 1/D and H, while the U was significantly lower than the NS-CK (*p* < 0.05). In MS, the 1/D and H of soil microorganisms treated with carbendazim (1 mg/kg and 2 mg/kg, MS-CBD) showed no significant difference compared with the MS-CK. The U of soil microorganisms in MS-CBD2 was not significantly different from the MS-CK, while the U in MS-CBD1 was significantly lower than that of the MS-CK (*p* < 0.05).

The changes in the functional diversity index of earthworm gut microorganisms under CBD stress are shown in Figures 6–8. The 1/D and H of earthworm gut microorganisms were suppressed in NS-CBD, but the differences were not significant (*p* < 0.05) when compared with the NS-CK. The U of earthworm gut microorganisms in NS-CBD2 was significantly lower than that in the NS-CK (*p* < 0.05), but there was no significant difference between NS-CBD1 and NS-CK. MS-CBD1 resulted in a significant reduction in the 1/D, H, and U indices of earthworm gut microorganisms compared to MS-CK (*p* < 0.05). The MS-CBD2 showed no significant difference in 1/D and H, while the U was significantly higher than that of the MS-CK (*p* < 0.05). The diversity indices of earthworm gut microorganisms in the MS-CBD2 were higher than those in the NS-CBD2.

## 4 Discussion

The acetylcholine receptor is the target of the triazole fungicide carbendazim, which can interfere with the signal transmission of the central nervous system of the body, causing paralysis or even death. Whereas AChE can hydrolyze acetylcholine to ensure normal neurotransmission and maintain normal function of the nervous system (Goulson and Kleijn, 2013). After exposure to carbendazim, the AChE activity in earthworms initially increased with contamination concentration but gradually decreased over time. Similar to our results, Yang et al. (2018) found that the AChE activity of earthworms increased significantly on the 3rd and 7th d of exposure to 3-(2-chloroethyl) phosphate (1 mg/kg and 10 mg/kg), and decreased significantly on the 14th d. Hackenberger et al. (2018) found that the AChE activity of earthworm (Dendrobaenaveneta) increased significantly after 7 d of exposure to 2,160 μg/kg glyphosate, and returned to the control level after 28 d. This is due to the fact that AChE activity gradually recovers in the later stages of staining because the pesticide has a lower comprehensive toxic effect with the extension of exposure time and the cells develop an anti-stress repair mechanism. In the MS, stress response and recovery of AChE activity were more rapid under the high concentration of pollution, because organic matter increased the nutrition of earthworms and promoted the growth and development of earthworms. Moreover, the intake of organic fertilizer increased the types and quantities of beneficial microorganisms in the earthworm gut of earthworms, enhanced the symbiotic network between microorganisms and earthworms, and improved the ability to resist external risks and stress (Markad et al., 2015; Chen et al., 2017). However, some studies have reported different results that the AChE activity of earthworms can be inhibited by some insecticides, such as triazophos, chlorpyrifos, deltamethrin, and so on (Bednarska et al., 2017; Singh et al., 2019). When earthworms were exposed to the neonicotinoid guadipyr initially, AChE activity was significantly reduced, but during subsequent tests, AChE activity returned to the control level without significant difference (Wang et al., 2015). In addition, Yang et al. (2018) found that trimethylphenyl phosphate (0.1 mg/kg and 10 mg/kg) significantly inhibited AChE activity on the 3rd d of exposure, and then AChE activity gradually recovered with the extension of exposure time. Zhao et al. (2019) found that N-ethyl perfluorooctane sulfonamide ethanol treatment had no significant effect on AChE activity of earthworms. Due to the various mechanisms of action and complex effects of pesticides, their effects on AChE activity are also different. Therefore, when using AChE as an environmental biomarker, especially in environments contaminated with multiple classes of chemicals, it is important to assess the effect of contaminants on AChE activity (Frasco et al., 2005).

CAT is an important antioxidant enzyme and a major defense enzyme against ROS in the body. CBD exposure significantly increased the CAT activity of earthworms, indicating that the antioxidant enzyme system of earthworms was activated in response to oxidative stress. Among them, SOD discriminates superoxide anion into H_2_O_2_, and CAT in turn catalyzes H_2_O_2_ into O_2_ and H_2_O (Lushchak, 2016), thus actively resisting oxidative damage caused by exogenous pollution. Similar to our results, different concentrations of fluoxastrobin (0.1, 1.0, and 2.5 mg/kg) in soil induced the changes in earthworm CAT activity, and the values for CAT were lower on days 7, 14, and 28 and greater on day 21 compared to those of the controls (Zhang et al., 2018). Xu et al. (2021) found that azoxystrobin (1.0 mg/kg and 2.5 mg/kg) in black soil and red clay soil induced a significant increase in earthworm CAT, which remained significantly higher than the control treatment after 56 d. Our results also revealed that CAT activity slowly decreased on the 14th day after pollutant exposure in earthworms but remained significantly higher than the control, indicating that the oxidation of the body stimulates the antioxidant capacity of CAT. The increase of hydroxyl free radicals caused by exposure to pollutants would enhance CAT activity, leading to oxidative stress (Malev et al., 2012). Zhu et al. (2020) found that the CAT activity of earthworms in red clay soil with 0.1 mg/kg and 1.0 mg/kg chlorpyrifos was higher than that in the control treatment during the whole exposure period (56 d). This may be because the dynamic balance of SOD-H_2_O_2_ did not negatively affect CAT activity. Liu et al. (2021) also found that the CAT activity of earthworms exposed to 20 mg/kg R-acetochlor was significantly higher than that in the control treatment within 7–42 d. In contrast to our findings, there was a trend of stimulation followed by recovery or inhibition of earthworm CAT activity under some pollutant stresses with longer exposure times. Yin et al. (2021) found that the CAT activity of *M.guillelmi* increased and then decreased significantly after 7 d of exposure to 0.1–50 mg/kg tetracycline. Under the stress of 10 mg/kg ciprofloxacin, the CAT activity of earthworms was higher on the 7th and 14th d and returned to the control level with the extension of treatment time (Yang et al., 2020).

The AWCD in the Biolog-ECO disk reflects the overall capacity of soil microorganisms to utilize carbon sources and microbial activity, and can be used as an effective indicator of soil microbial activity, which is sensitive to soil environmental stress (O’Donnell et al., 2001; Han et al., 2020). The AWCD values of microorganisms in the MS were higher than that in the NS, which may be due to the fact that manure not only contains a large number of microorganisms, but also provides a large amount of carbon, nitrogen, and other nutrients for the growth and propagation of soil microorganisms (Fang et al., 2014; Han et al., 2019; Zeb et al., 2020). Similarly, Lin et al. (2016) reported that two earthworm species, *Eisenia fetida* and *Amynthas robustus* E. Perrier, stimulated the soil microbial utilization of amines, amino acids, carbohydrates, and carboxyl acids in pentachlorophenol-contaminated soils. The AWCD values of the CBD2 were close to those of the control soil, which may be attributed to the fact that the higher concentration of carbendazim (2 mg/kg) stimulated the proliferation of more versatile microbial populations in the gut of the earthworms. This, in turn, affects the community structure of soil microorganisms around the earthworms and promoting the overall activity of soil microorganisms to maintain a high level (Han et al., 2023). The NS-CBD2 can stimulate the adaptation of some microorganisms in soil at the absence of nutrients. The change rule of AWCD values in earthworm gut microorganisms was more complicated, but the AWCD values of earthworm gut microorganisms under the MS-CBD2 were significantly increased. This increase is because organic fertilizer can provide essential nutrients for earthworms, and the CBD-enriched residues in the body stimulate more functional bacteria in the gut, which can enhance the activity in the intestinal tract and optimize the earthworms’ ecological service function (Lin et al., 2018; Xiao et al., 2020; Han et al., 2023). Ning et al. (2019) studied the changes in the functional diversity of earthworm gut and soil microbial communities under cadmium stress by the Biolog method. It was found that earthworms not only regulate their physiological functions (such as microbial community structure and stress mechanism), but also influence the external soil microbial community structure to obtain the substances required for growth.

The diversity indices actually reflect different aspects of the functional diversity of soil and gut microbial communities, with 1/D representing the most common dominant species in the community, H evaluating the community species richness, and U being a measure of community species homogeneity. The microbial 1/D, H, and U of MS treatments in soil were higher than those of NS treatments as a whole, indicating that organic fertilizer additions increased soil microbial dominance, abundance and homogeneity. Similar to our results, Urbaniak et al. (2024) showed that the implementation of NBS (Wild flower Meadow) had a positive influence on the values of Shannon-Weaver diversity (H′) in spring, H′ increased by 63% compared to pre-implementation stage. The substrate richness index (S) increased by 53% after NBS implementation in the spring season, while S values were lower in other locations. The change rule of earthworm gut microbial diversity indices in the MS was similar to that of soil microbial diversity index. With the increase of carbendazim concentration, microbial activity showed inhibition first and then increased, which is consistent with the previous research results (Fang et al., 2014; Han et al., 2019). Meanwhile, the microbial diversity indices in both gut and soil of the MS-CBD2 were higher than those in the NS-CBD2. This may be attributed to the fact that most of the microorganisms carried by manure were able to colonize the gut of earthworms, resulting in microbial community compositions of the gut microorganisms that were similar to those of the surrounding soil microorganisms to a certain extent (Gao et al., 2022; Lejoly et al., 2023; Ferlian et al., 2024), and such an effect has also been reported for CBD and glyphosate (Ratcliff et al., 2006; Tortella et al., 2013). Zhou et al. (2020) found that the Simpson index and Shannon Wiener index of earthworm gut microorganisms initially increased and then decreased with the increase of cadmium concentration, indicating that the richness and dominance of earthworm gut microbial community were significantly enhanced by pollutant within a certain concentration range. However, there was a significant difference between the gut microbial diversity index of earthworm and the soil microbial diversity index in the NS, indicating that the addition of organic fertilizers provides nutrients for microbial growth and reproduction, promotes the proliferation of functional gut bacteria, increases the diversity and function of soil microorganisms when they enter the soil, and promotes the remediation of pollutants together with the soil microorganisms, making the soil environment more stable (Vivas et al., 2009; Yakushev et al., 2009; Doan et al., 2013). At the same time, earthworms and their companion animals produce more secretions accompanied by humus production, which enriches the food chain in the ecosystem (Hale et al., 2005; Han et al., 2023).

## 5 Conclusion

The study focused on the enzyme activities and gut microbial of earthworms response to CBD under the treatment of manure. The results showed that the AChE activities of earthworms in NS-CBD were stimulated at the initial stage and significantly inhibited at the later stage with the extension of exposure time. The trends of earthworm AChE activity under MS-CBD were generally similar to those in NS-CBD, except that the stimulatory and inhibitory effects on earthworm AChE activity were advanced by the treatment of MS-CBD2. The trend of CAT activity of earthworms in MS was similar to that in NS, and the CBD treatment resulted in a tendency for CAT activity to initially increase and then remain at a higher activity. The AWCD values, 1/D, and H of earthworm gut microorganisms did not change significantly in the NS, but MS-CBD2 resulted in significant increase in AWCD values, 1/D, H, and U of earthworm gut microorganisms. The dominance, abundance, and homogeneity of earthworm gut microorganisms in the MS-CBD2 were significantly higher than those in the NS-CBD2. The results of this study can provide a data reference and theoretical basis for monitoring and remediation of pesticide pollution in soil environment.

## Data Availability

The raw data supporting the conclusions of this article will be made available by the authors, without undue reservation.
